# Deprescribing proton pump inhibitors in long-term care: Outcomes of a pharmacist-led initiative

**DOI:** 10.1016/j.rcsop.2026.100820

**Published:** 2026-07-03

**Authors:** Amira Muhana, Rizah Anwar Assadi, Amir Mahdi, Alaa Fathy, Martin Farina, Mariam Jihad Diab

**Affiliations:** a Amana Healthcare, United Arab Emirates; b The Life Corner, United Arab Emirates; c Dubai Health, United Arab Emirates; d Dubai Medical University, United Arab Emirates

## Abstract

**Background:**

Proton pump inhibitors (PPIs) are frequently continued without clear indication in long-term care (LTC) settings. Evidence-based deprescribing strategies may reduce unnecessary exposure, but real-world data from Middle Eastern LTC facilities remain limited.

**Objective:**

To evaluate the feasibility, implementation, and short-term safety of a pharmacist-led PPI deprescribing initiative in a long-term care facility.

**Methods:**

This retrospective cohort study included LTC residents receiving PPIs who underwent pharmacist-led medication review between September and November 2024. Residents were classified eligible or ineligible for deprescribing using guideline-aligned criteria. Deprescribing required approval from the attending physician and resident or family. Outcomes included deprescribing implementation, maintenance off PPIs at 3-month follow-up, symptom recurrence, PPI reinitiation, and gastrointestinal (GI) bleeding events.

**Results:**

Among 68 residents, 35 (51.5%) were eligible for deprescribing. Deprescribing was implemented in 26 eligible residents (74.3%; 95% CI 56.7–87.5). Sustained PPI discontinuation occurred in 25 of 35 eligible residents (71.4%; 95% CI 53.7–85.4). Among the 26 residents who discontinued therapy, 25 (96.2%; 95% CI 80.4–99.9) remained off PPIs at 3 months. One resident (3.8%; 95% CI 0.1–19.6) had GI bleeding during follow-up. No other adverse events were observed, and no resident required rescue acid-suppressive therapy or urgent evaluation.

**Conclusion:**

A pharmacist-led deprescribing initiative was feasible to implement in a long-term care facility and was associated with high deprescribing uptake and maintenance off therapy among eligible residents. These findings support the potential role of pharmacist-led deprescribing in LTC settings and highlight opportunities for further evaluation of deprescribing programs in underrepresented regions.

## Introduction

Proton pump inhibitors (PPIs) are among the most frequently prescribed medications worldwide, with long-term use often extending beyond evidence-based indications. Recent reviews have documented high rates of potentially inappropriate PPI prescribing in older adults, including prolonged exposure without clear justification.^1^ Similar patterns have been observed across hospital and ambulatory settings worldwide, where inappropriate continuation is frequently noted during care transitions and prolonged chronic therapy.^2^, ^3^ Evidence indicates that PPI overuse is widespread, with high rates reported among U.S. veterans^4^ and across European populations.^5^ Regionally, a study from Lebanon reported substantial PPI overuse in Middle Eastern adults, highlighting the global nature of this issue.^6^

Although PPIs have a favorable short-term safety profile, accumulating evidence has raised concerns regarding potential adverse effects associated with long-term use. Meta-analyses have identified associations between chronic PPI exposure and increased fracture risk^7^ and higher all-cause mortality among older adults.^8^ While these findings remain subject to confounding and do not confirm causality, they highlight the importance of minimizing unnecessary prolonged therapy. Accordingly, current clinical guidelines advocate routine reassessment of PPI indication and active deprescribing for patients without ongoing evidence-based need.

Evidence-based deprescribing frameworks, including the 2022 American Gastroenterological Association (AGA) Clinical Practice Update^9^ and the Canadian PPI deprescribing guideline,^10^ advise discontinuation, tapering, or step-down therapy for patients who have completed short-term treatment for uncomplicated gastroesophageal reflux disease (GERD) or mild esophagitis and no longer require acid suppression. Both guidelines also recommend counselling patients about the potential for rebound acid hypersecretion following abrupt discontinuation.^11^ In addition, they outline explicit clinical scenarios in which deprescribing should generally be avoided, such as a history of upper gastrointestinal bleeding, severe (Los Angeles grade C/D) erosive esophagitis, or the need for gastroprotection in high-risk antithrombotic regimens.

The effectiveness of deprescribing interventions in reducing inappropriate PPI use has been demonstrated across a wide range of care settings. Randomized and observational studies indicate that structured deprescribing strategies are feasible and associated with favorable outcomes in both older adults and mixed-age populations.^12^, ^13^ Pharmacist-led deprescribing has emerged as particularly impactful: randomized trials and feasibility studies consistently report improvements in medication appropriateness and reductions in unnecessary PPI use in hospital and long-term care environments.^14^, ^15^ Collectively, this evidence highlights the value of integrating pharmacists into team-based deprescribing initiatives and underscores their role in operationalizing guideline-directed medication review.

Despite growing interest in deprescribing, research within long-term care (LTC) settings remains sparse. The majority of existing evidence originates from primary care or inpatient populations, with relatively few studies conducted in LTC facilities and none, to our knowledge, reported from the Middle East. A pharmacist-led LTC deprescribing study conducted in Canada demonstrated feasibility and favorable outcomes,^16^ but evidence remains limited across diverse geographic contexts. To address this gap, we conducted a retrospective evaluation of a pharmacist-led PPI deprescribing initiative in a long-term care facility, assessing deprescribing implementation, maintenance off therapy, and short-term safety outcomes.

## Methodology

### Study design and setting

This study was a retrospective cohort study conducted at a long-term care (LTC) facility in the United Arab Emirates (UAE). The study evaluated the outcomes of a pharmacist-led proton pump inhibitor (PPI) deprescribing initiative implemented between September and November 2024. All data were derived from routinely collected electronic medical records (EMRs) and medication administration records (MARs). The facility provides continuous multidisciplinary care to residents with chronic medical conditions, including predominantly older adults as well as younger individuals requiring long-term institutional care.

Data collection and reporting followed established methodological standards for observational research. Ethical approval for this study was granted by the Amana Healthcare Research Ethics Committee (REC), approval number RA-020, with approval valid from May 2025 to May 2027.

### Eligibility and patient classification

All residents with an active PPI prescription who were reviewed by the clinical pharmacist during the study period were assessed for deprescribing eligibility. Eligibility was determined using clinical criteria derived from the 2022 AGA Clinical Practice Update and the Canadian PPI deprescribing guideline, with consideration of each resident's documented indication, symptom history, and gastrointestinal bleeding risk profile.

Eligibility assessment followed a structured review process based on recommendations from the 2022 American Gastroenterological Association Clinical Practice Update and the Canadian evidence-based PPI deprescribing guideline. For each resident, the clinical pharmacist reviewed the documented indication for PPI therapy, recognizing that the commonly documented indication of ‘GI prophylaxis’ often lacked sufficient detail to establish a guideline-supported need for long-term therapy. In such cases, eligibility was determined through individualized assessment of gastrointestinal bleeding risk factors, clinical history, and concomitant medications. Residents were considered eligible for deprescribing when no ongoing guideline-supported indication for chronic PPI therapy was identified. A summary of the eligibility assessment criteria is provided in Supplementary Table S1.

Residents were categorized into two groups:

1. **Eligible for deprescribing** – those without a guideline-supported indication for continued PPI therapy (e.g., resolved short-term indications or use for non-specific prophylaxis without high-risk features).
2. **Not eligible** – those with guideline-defined indications for ongoing therapy, such as prior upper gastrointestinal bleeding, severe erosive esophagitis, or need for gastroprotection with high-risk antithrombotic regimens.

**Fig. 1:**
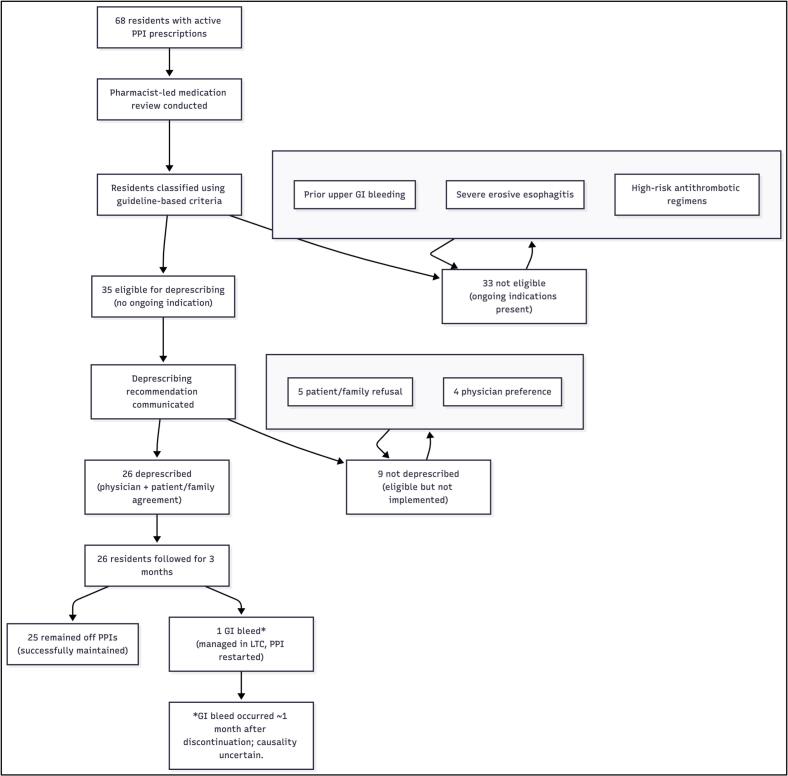
Study flow diagram illustrating resident eligibility assessment, deprescribing implementation, and 3-month follow-up outcomes.

A summary of resident classification and deprescribing implementation is presented in Fig. 1.

### Pharmacist-led deprescribing intervention

A clinical pharmacist conducted structured medication reviews as part of routine multidisciplinary clinical rounds involving physicians, pharmacists, nurses, and other healthcare professionals. During these reviews, the pharmacist assessed each resident's documented indication for PPI therapy, duration of treatment, gastrointestinal bleeding risk factors, relevant comorbidities, and concomitant medications to determine eligibility for deprescribing according to established guideline recommendations.

For residents identified as potential candidates for deprescribing, recommendations were documented in the electronic medical record and discussed directly with the attending physician during multidisciplinary rounds. Physicians retained full discretion to accept or decline recommendations, and deprescribing was implemented only after physician approval and agreement from the resident or family when applicable.

The reviews were conducted by experienced licensed clinical pharmacists who were board-certified specialist pharmacists practicing in Abu Dhabi, United Arab Emirates.

For accepted recommendations, PPIs were discontinued abruptly; no tapering protocols or step-down therapy to alternative acid-suppressive agents were used during the study period. Residents subsequently underwent routine clinical monitoring by nursing and medical staff, with documentation of gastrointestinal symptoms, adverse events, and any need for PPI reinitiation during the 3-month follow-up period.

Residents were monitored during routine clinical care at approximately 4 and 12 weeks following deprescribing. Monitoring was performed by nursing and medical staff as part of ongoing clinical assessments and multidisciplinary rounds. For residents able to communicate verbally, symptom monitoring focused on heartburn, regurgitation, dyspepsia, and epigastric pain. For residents with limited verbal communication, staff monitored for potential indicators of gastrointestinal discomfort, including reduced appetite, weight loss, and agitation. No validated symptom assessment tool was used; symptom ascertainment relied on routine clinical documentation within the electronic medical record.

### Data sources and variables

Data were obtained from the facility's electronic medical record (EMR) system and the medication administration record (MAR). A structured data abstraction sheet was developed for the study, and a single clinical pharmacist extracted all variables to ensure consistency. No missing data were identified for any variable.

Collected variables included resident demographics (age, sex), documented PPI indication, duration of PPI therapy, and relevant comorbidities or medications associated with gastrointestinal bleeding risk (e.g., antiplatelets, anticoagulants, NSAIDs, systemic corticosteroids). For eligible residents, we recorded whether deprescribing was implemented and, when not implemented, the documented reason (physician preference or patient/family refusal).

Follow-up variables included any documented gastrointestinal symptoms, PPI reinitiation, and adverse events, specifically clinically suspected gastrointestinal bleeding event during the 3-month follow-up period.

### Outcome definitions

The primary outcome was sustained PPI discontinuation, defined as complete discontinuation of PPI therapy without reinitiation during the 3-month follow-up period.

Secondary outcomes included symptom recurrence, and safety outcomes included clinically suspected gastrointestinal bleeding occurring during follow-up.

PPI reinitiation was defined as any documented restart of a PPI order, any PPI dose administered, or re-entry of a PPI into the medication administration record.

Symptom recurrence was defined as any documented reflux-like or dyspepsia-like symptom, including patient-reported symptoms or nurse/physician-observed signs of discomfort in residents unable to communicate verbally. Mild, self-limited symptoms were recorded but were not considered treatment failures unless they resulted in PPI reinitiation.

Gastrointestinal (GI) bleeding was identified based on a physician-documented diagnosis supported by objective clinical findings, including melena, hematemesis, positive fecal occult blood testing, and/or a clinically significant decline in hemoglobin consistent with suspected GI blood loss. When available, findings from diagnostic investigations were reviewed to further characterize the event.

### Statistical analysis

Data were analyzed using IBM SPSS Statistics (version 29). Categorical variables were summarized as frequencies and percentages. Group comparisons were performed using Pearson's chi-square test or Fisher's exact test when expected cell counts were < 5.

Key proportions—including deprescribing implementation, sustained discontinuation, symptom recurrence, maintenance off therapy, and GI bleeding—were reported with 95% exact binomial confidence intervals. The association between guideline-defined eligibility and actual deprescribing was summarized using a 2 × 2 table with an odds ratio calculated using a continuity correction.

No multivariable analyses were conducted due to the small sample size and very low event rate. No missing data were identified for any study variable. A *p*-value <0.05 was considered statistically significant.

## Results

### Baseline characteristics

A total of 68 residents were included in the evaluation (Fig. 1). The median age category was 80–89 years, and 47.1% were female. Most residents (82.4%) were receiving a PPI for gastrointestinal prophylaxis, and 63.2% had been on PPI therapy for more than six months. Pantoprazole 40 mg once daily was the most frequently prescribed regimen (58.8%), followed by esomeprazole 40 mg once daily (33.8%). Baseline characteristics are summarized in Table 1.

**Table 1:** Baseline characteristics of residents.

Demographics and baseline parameters	No. of patients (%)
Sex	
Female	32 (47.1)
Male	36 (52.9)

Age (years)	
18–19	2 (2.9)
20–29	3 (4.4)
30–39	2 (2.9)
40–49	3 (4.4)
50–59	5 (7.4)
60–69	5 (7.4)
70–79	17 (25.0)
80–89	19 (27.9)
90–99	12 (17.6)

PPI indication	
Unspecified / blank	1 (1.5)
Documented bleeding GI ulcer	8 (11.8)
Gastritis	1 (1.5)
GI prophylaxis	56 (82.4)
High risk of bleeding	2 (2.9)

PPI use (total daily dose)	
Pantoprazole 20 mg once daily	1 (1.5)
Pantoprazole 40 mg once daily	40 (58.8)
Esomeprazole 20 mg once daily	4 (5.9)
Esomeprazole 40 mg once daily	23 (33.8)

Length of stay	
< 1 month	1 (1.5)
1–3 months	4 (5.9)
3–6 months	13 (19.1)
> 6 months	50 (73.5)

Duration of PPI treatment	
< 1 month	2 (2.9)
1–3 months	10 (14.7)
3–6 months	13 (19.1)
> 6 months	43 (63.2)

**Concurrent medications**	
Anticoagulants	43 (63.2)
Antiplatelets	13 (39.4)
Steroids	4 (12.1)

### Guideline-based eligibility and implementation

Based on guideline-aligned criteria, 35 of 68 residents (51.5%) were classified as eligible for deprescribing. The remaining 33 residents (48.5%) had ongoing indications for continued PPI therapy, most commonly prior upper gastrointestinal bleeding, severe erosive esophagitis, or high-risk antithrombotic regimens. Eligibility classification is summarized in Fig. 1.

Of the 35 eligible residents, 26 (74.3%; 95% CI 56.7–87.5) underwent deprescribing. Deprescribing was not implemented in 9 eligible residents (25.7%), primarily due to patient or family refusal (5 residents; 56%) or physician preference (4 residents; 44%). Comparative characteristics of eligible versus ineligible residents are shown in Table 2.

**Table 2:** Comparison of baseline characteristics between residents eligible and not eligible for PPI deprescribing.

Characteristic	Fit Criteria (*n* = 35)	Did Not Fit Criteria (*n* = 33)	*p*-value
**Age group**			0.238
18–19	2 (5.7%)	0 (0%)	
20–29	3 (8.6%)	0 (0%)	
30–39	1 (2.9%)	1 (3.0%)	
40–49	2 (5.7%)	1 (3.0%)	
50–59	4 (11.4%)	1 (3.0%)	
60–69	3 (8.6%)	2 (6.1%)	
70–79	6 (17.1%)	11 (33.3%)	
80–89	7 (20.0%)	12 (36.4%)	
90–99	7 (20.0%)	5 (15.2%)	

**Sex**			0.092
Male	22 (62.9%)	14 (42.4%)	
Female	13 (37.1%)	19 (57.6%)	

**PPI dose**			0.165
Pantoprazole 20 mg OD	1 (2.9%)	0 (0%)	
Pantoprazole 40 mg OD	19 (54.3%)	21 (63.6%)	
Esomeprazole 20 mg OD	4 (11.4%)	0 (0%)	
Esomeprazole 40 mg OD	11 (31.4%)	12 (36.4%)	

**Reason for PPI**			0.009
GI prophylaxis	35 (100%)	21 (63.6%)	
History of GI bleed	0 (0%)	8 (24.2%)	
Gastritis	0 (0%)	1 (3.0%)	
High risk of bleeding	0 (0%)	2 (6.1%)	
Other/blank	0 (0%)	1 (3.0%)	

Concurrent medications			–
Anticoagulants	20 (57.1%)	23 (69.7%)	0.283
Antiplatelets	0 (0%)	13 (39.4%)	<0.001
Steroids	0 (0%)	4 (12.1%)	0.034

**Other reasons**			–
Patient refusal	5 (14.3%)	0 (0%)	0.024
Physician decision	4 (11.4%)	1 (3.0%)	0.185
Patient-related	0 (0%)	4 (12.1%)	0.034

**Length of stay (coded)**			0.161
< 1 month	1 (2.9%)	0 (0%)	
1–3 months	1 (2.9%)	3 (9.1%)	
3–6 months	4 (11.4%)	9 (27.3%)	
> 6 months	29 (82.9%)	21 (63.6%)	

**Duration of PPI treatment**			0.564
< 1 month	2 (5.7%)	0 (0%)	
1–3 months	5 (14.3%)	5 (15.2%)	
3–6 months	6 (17.1%)	7 (21.2%)	
> 6 months	22 (62.9%)	21 (63.6%)	

### Primary outcome: sustained PPI discontinuation

Among the 35 residents who met guideline-based criteria for deprescribing, 25 achieved sustained PPI discontinuation throughout follow-up, corresponding to 71.4% (95% CI 53.7–85.4). Of the 26 residents whose PPI was discontinued, 25 (96.2%; 95% CI 80.4–99.9) remained off therapy throughout the 3-month follow-up period.

### Secondary and safety outcomes

No residents reported or exhibited documented gastrointestinal symptoms following discontinuation.

One resident experienced a physician-documented gastrointestinal bleeding event approximately one month after deprescribing. No causal relationship between the event and deprescribing could be established. No additional adverse events occurred during follow-up.

The resident developed melena, a positive fecal occult blood test, and a decline in hemoglobin from 9.3 g/dL to 7.7 g/dL approximately one month after PPI discontinuation. Colonoscopy identified colonic polyps but did not reveal an active bleeding source or lesion requiring intervention. Although an upper gastrointestinal source was clinically suspected because of the presence of melena, no definitive source of bleeding was identified. The resident was managed conservatively with reinitiation of pantoprazole 40 mg twice daily and remained clinically stable thereafter.

No resident required rescue acid-suppressive therapy, urgent clinical evaluation, hospitalization, or escalation of care. No new or unexplained anemia was identified during the follow-up period.

### Comparative analyses

Residents eligible for deprescribing differed from those deemed ineligible in several clinically coherent ways. Eligible residents were more likely to have been prescribed PPIs for gastrointestinal prophylaxis without ongoing high-risk features, whereas ineligible residents more frequently had prior gastrointestinal bleeding, severe erosive esophagitis, or high-risk antithrombotic regimens.

Use of antiplatelet agents and systemic corticosteroids, which are key contributors to high gastrointestinal bleeding risk, was confined to the ineligible group. Anticoagulant monotherapy did not differ significantly between groups, consistent with the study's individualized bleeding-risk assessment. No significant differences were observed in length of stay or duration of PPI therapy. Full comparative characteristics are presented in Table 2.

## Discussion

This retrospective evaluation found that over half of LTC residents were eligible for PPI deprescribing, and most remained off therapy at 3 months. Only one gastrointestinal bleeding event occurred, with unclear causality. Overall, pharmacist-led deprescribing was feasible to implement in this setting and was associated with favorable short-term outcomes, although the study design does not permit definitive conclusions regarding effectiveness or safety.

Our findings are broadly consistent with previous deprescribing studies reporting reductions in unnecessary PPI use following structured deprescribing interventions across diverse clinical settings. Prior work in outpatient and inpatient populations has demonstrated that most eligible patients can discontinue PPIs successfully and remain off therapy at short-term follow-up.^12^, ^13^ Pharmacist-led interventions have similarly achieved favorable outcomes in hospitals and long-term care facilities, including improved medication optimization and high rates of successful discontinuation.^14^, ^15^ The deprescribing success observed in our study is also consistent with the feasibility reported in the Canadian long-term care study by Tandun et al. (2019),^16^ suggesting that pharmacist-led deprescribing can be implemented effectively across different care models.

The high rate of maintenance off PPIs in our cohort suggests that many residents receiving chronic acid suppression do not require ongoing therapy and can discontinue treatment when appropriately selected. Notably, abrupt discontinuation, without tapering or step-down therapy, was well tolerated in this population. The decision to implement abrupt discontinuation was based on multidisciplinary consensus among the clinical pharmacy team, lead physician, and consultant geriatrician, and was consistent with available deprescribing guidance. Although rebound acid hypersecretion is a recognized concern following PPI withdrawal, evidence comparing discontinuation strategies remains limited. In a randomized study comparing abrupt discontinuation with tapering, no significant difference was observed in long-term successful PPI discontinuation at 12 months, although patients in the tapering group experienced fewer symptoms during follow-up.^17^ These findings suggest that both approaches are reasonable options, and abrupt discontinuation was selected as a pragmatic and standardized strategy for implementation within our long-term care setting.

The single gastrointestinal bleeding event observed during follow-up warrants careful interpretation. The resident presented with melena, a positive fecal occult blood test, and a clinically significant decline in hemoglobin; however, subsequent investigation did not identify a definitive bleeding source. Although an upper gastrointestinal origin was considered clinically plausible, this remained unconfirmed, and a causal relationship between PPI discontinuation and the event could not be established. Consequently, the event should be interpreted as a safety signal occurring after deprescribing rather than evidence that deprescribing directly caused gastrointestinal bleeding. Given the retrospective design and the complexity of this medically frail long-term care population, causal attribution of individual adverse events remains inherently limited.

Notably, no gastrointestinal symptoms were documented following PPI discontinuation despite the well-described phenomenon of rebound acid hypersecretion after PPI withdrawal. While this finding may reflect successful patient selection and close clinical monitoring, it may also be influenced by limitations in symptom ascertainment. Because symptom monitoring relied on routine clinical documentation rather than a validated assessment instrument, mild or transient symptoms may not have been consistently captured, particularly among residents with impaired cognition or communication difficulties.

These findings suggest that, with appropriate patient selection and routine clinical monitoring, pharmacist-led deprescribing may be implemented in long-term care practice without major short-term complications; however, larger controlled studies are needed to more definitively evaluate safety.

The results of this initiative also highlight the pivotal role of pharmacists in deprescribing within long-term care. Pharmacists are uniquely trained to perform detailed medication assessments, apply guideline-based criteria consistently, and identify residents who no longer require chronic therapies. Their involvement facilitates evidence-based decision-making and reduces therapeutic inertia, which is particularly important in LTC settings where polypharmacy is prevalent and prescribers may have limited time for comprehensive review. Pharmacists also contribute to patient and family engagement, addressing concerns regarding symptom recurrence and providing reassurance about the deprescribing process. The high implementation rate observed in our study supports the value of integrating pharmacists as core members of deprescribing teams.

This study has several strengths, including its real-world long-term care setting, use of guideline-based criteria for eligibility, and comprehensive follow-up with no missing data. The pharmacist-led review ensured consistent application of deprescribing criteria, and all residents remained within the facility throughout follow-up, allowing continuous monitoring for symptoms and adverse events.

However, several limitations should be considered. The study was conducted at a single center with a modest sample size, which limits generalizability. The retrospective design introduces potential information and documentation bias, particularly with respect to symptom capture. Although residents underwent routine clinical and nursing follow-up, no standardized symptom assessment instrument was used. Consequently, mild, transient, or self-limited reflux symptoms may have been underreported in the medical record. This limitation is especially relevant for residents with cognitive impairment, communication difficulties, or limited ability to report symptoms directly, in whom rebound acid-related symptoms may have been more difficult to detect. Therefore, the absence of documented gastrointestinal symptoms should be interpreted cautiously. In addition, all data abstraction was performed by a single clinical pharmacist. Although the use of a structured data collection form helped promote consistency, the absence of independent duplicate data extraction may have introduced the potential for information bias or data abstraction errors.

The very low event rate precluded multivariable adjustment. In addition, the 3-month follow-up period limits assessment of longer-term outcomes after deprescribing. Although most residents remained off therapy during follow-up, recurrence of reflux-related symptoms, delayed need for acid-suppressive therapy, or gastrointestinal complications may occur beyond the observation period and therefore may not have been captured. Consequently, the sustainability of deprescribing success and the long-term safety profile of PPI discontinuation cannot be fully assessed from these data. Future studies with extended follow-up are needed to better characterize long-term clinical outcomes following deprescribing in long-term care populations.

The generalizability of these findings should also be considered carefully. This study was conducted within a single long-term care facility operating under a specific multidisciplinary care model, and local prescribing practices, deprescribing culture, healthcare resources, and resident characteristics may differ from those of other institutions. In addition, long-term care facilities remain relatively underrepresented in the Middle Eastern literature, and published evidence regarding deprescribing interventions in these settings is limited. Consequently, the applicability of our findings to other long-term care environments should be interpreted cautiously until confirmed by larger multicenter studies across diverse healthcare systems.

These findings have several implications for long-term care practice. First, they demonstrate the feasibility of integrating pharmacist-led deprescribing into routine workflows, with high physician acceptance and sustained discontinuation rates. Addressing patient and family hesitancy—identified as the most frequent barrier—may further improve deprescribing uptake and suggests the value of structured education and shared decision-making. Finally, as deprescribing research in long-term care remains limited, particularly in the Middle East, larger multicenter evaluations and prospective designs are needed to better characterize long-term outcomes, compare deprescribing strategies, and understand resident and caregiver perspectives. Although this initiative focused specifically on PPIs, medication optimization and deprescribing are routinely addressed during multidisciplinary medication reviews, and PPIs represented one component of a broader effort to improve medication appropriateness in long-term care residents.

## Conclusion

In summary, this study demonstrates the feasibility of implementing a pharmacist-led PPI deprescribing initiative in a long-term care facility. Most eligible residents underwent deprescribing and remained off therapy during short-term follow-up, with few documented adverse events. These findings support the potential role of pharmacists in promoting guideline-concordant medication use in long-term care and provide preliminary regional data to inform future prospective and controlled studies evaluating deprescribing outcomes.

## CRediT authorship contribution statement

**Amira Muhana:** Writing – original draft, Methodology, Data curation, Conceptualization. **Rizah Anwar Assadi:** Writing – review & editing, Writing – original draft, Formal analysis. **Amir Mahdi:** Methodology, Data curation, Conceptualization. **Alaa Fathy:** Methodology, Conceptualization. **Martin Farina:** Methodology, Conceptualization. **Mariam Jihad Diab:** Writing – review & editing, Writing – original draft, Supervision.

## Declaration of competing interest

The authors declare that they have no known competing financial interests or personal relationships that could have appeared to influence the work reported in this paper.

## Appendix A Supplementary data

Supplementary material Supplementary Table S1. Guideline-Based Criteria Used to Determine Eligibility for Proton Pump Inhibitor (PPI) Deprescribing During Clinical Pharmacist Medication Review
