# Supplementary material for: Deprescribing proton pump inhibitors in long-term care: Outcomes of a pharmacist-led initiative
Source: Explor Res Clin Soc Pharm. 2026 Jul 3;23:100820. doi: 10.1016/j.rcsop.2026.100820 (PMC13382033; doi:10.1016/j.rcsop.2026.100820)
Supplement: Supplementary material — Supplementary Table S1. Guideline-Based Criteria Used to Determine Eligibility for Proton Pump Inhibitor (PPI) Deprescribing During Clinical Pharmacist Medication Review [file mmc1.docx]

Supplementary Table S1. Guideline-Based Criteria Used for Eligibility Assessment of Proton Pump Inhibitor (PPI) Deprescribing

| **Clinical Scenario Assessed During Medication Review** | **Eligible for Deprescribing** | **Not Eligible for Deprescribing** |
| --- | --- | --- |
| PPI prescribed for non-specific "GI prophylaxis" without documented ongoing indication | ✓ |  |
| Previous short-term indication completed (e.g., resolved dyspepsia or uncomplicated GERD) | ✓ |  |
| No documented history of upper gastrointestinal bleeding | ✓ |  |
| No requirement for gastroprotection based on current medication regimen and bleeding risk assessment | ✓ |  |
| Previous upper gastrointestinal bleeding |  | ✓ |
| Severe erosive esophagitis (Los Angeles Grade C or D) |  | ✓ |
| Barrett’s esophagus |  | ✓ |
| Requirement for ongoing gastroprotection due to high-risk antithrombotic therapy and/or gastrointestinal bleeding risk factors |  | ✓ |
| Other physician-documented indication requiring long-term acid suppression |  | ✓ |

***Abbreviations:*** *GERD, gastroesophageal reflux disease; GI, gastrointestinal; PPI, proton pump inhibitor.*

**Note:** Eligibility assessments were performed by a clinical pharmacist using recommendations from the 2022 American Gastroenterological Association (AGA) Clinical Practice Update and the Canadian Evidence-Based Clinical Practice Guideline for Deprescribing Proton Pump Inhibitors. Final eligibility determinations incorporated individual clinical assessment, gastrointestinal bleeding risk factors, concomitant medications, and physician judgment.
